# Frail2Fit study: it was feasible and acceptable for volunteers to deliver a remote health intervention to older adults with frailty

**DOI:** 10.1016/j.tjfa.2025.100092

**Published:** 2025-10-03

**Authors:** SJ Meredith, L Holt, J Varkonyi-Sepp, A Bates, KA Mackintosh, MA McNarry, S Jack, J Murphy, MPW Grocott, SER Lim

**Affiliations:** a NIHR Applied Research Collaboration Wessex and Academic Geriatric Medicine, University of Southampton, Faculty of Medicine, Southampton, UK; b Faculty of Medicine, University of Southampton, Southampton, UK; c Hampshire and Isle of Wight Healthcare NHS Foundation Trust, Hampshire, UK; d University Hospital Southampton NHS Foundation Trust, Southampton, UK; e Perioperative and Critical Care Theme, NIHR Southampton Biomedical Research Centre, University Hospital Southampton NHS Foundation Trust / University of Southampton, Southampton, UK; f Applied Sports, Technology, Exercise and Medicine (A-STEM) Research Centre, Department of Sport and Exercise Sciences, Swansea University, Swansea, UK; g Faculty of Health and Social Sciences, Bournemouth University, Bournemouth, UK

**Keywords:** Older people, Rehabilitation, Deconditioning, Exercise, Nutrition

## Abstract

**Background:**

Physical activity (PA) and good nutrition are key to maintaining independence and reversing frailty among older adults.

**Objective:**

To evaluate the feasibility and acceptability of training volunteers to deliver a remote multimodal intervention to older people with frailty after hospital discharge.

**Design:**

Quasi-experimental mixed-methods feasibility study.

**Setting, and Participants:**

Twenty-seven older adults (mean age 80 years, 15 female) with frailty (Clinical Frailty Status ≥5) were enrolled from a National Health Service South England hospital, UK.

**Intervention:**

Volunteers were trained to deliver a 3-month intervention, comprising exercise, behaviour change, and nutrition guidance in a group, or one-to-one, using telephone or online platforms.

**Measurements:**

Feasibility was assessed by determining the number of volunteers recruited, trained, and retained; participant recruitment; and intervention adherence. Interviews were conducted with 16 older adults, 1 carer, and 5 volunteers to explore intervention acceptability, and were analysed using reflexive thematic analysis. Secondary health outcomes, such as physical activity and appetite, were compared at baseline, post-intervention, and follow-up (3-months).

**Results:**

The intervention was safe and acceptable to volunteers, and older adults with frailty. Five volunteers (mean age 16 years, 3 female) completed training, and 60 % (*n* = 3) were retained. Seventeen participants completed the intervention (8 online; 9 telephone). Participants attended 75 % (IQR 38–92) online sessions, and 80 % (IQR 68.5–94.5) telephone support. Volunteers provided emotional support to older people with frailty and encouraged health behaviour change. Older people learnt from each other’s shared experiences in the online group, and telephone calls provided them with reassurance and monitoring. Key components to success were volunteers received comprehensive training and support from a health practitioner, while key barriers were that participants lacked social support and experienced exercise discomfort.

**Conclusion:**

With appropriate training and support, volunteers can safely deliver a remote multimodal intervention for older adults with frailty following discharge from hospital.

## 1 Introduction

Approximately 47 % of older people in hospital aged over 65 are affected by frailty [1]. Frailty is characterised by a cumulative decline in biological reserves leading to impaired homoeostatic recovery following stressor events [2]. It is associated with increased risk of post-hospitalisation, disability, and mortality [[3], [4], [5]]. Moreover, the prevalence of deconditioning during hospital admission impacts significantly on the ability for older adults to maintain independence [6]. Key interventions for frailty management and to address deconditioning post-hospitalisation include exercise, and nutrition support, underpinned by behaviour change strategies [[7], [8], [9]]. However, in practice, access to models of care, such as multidisciplinary reablement services to support older people on discharge, is restricted by the health system’s capacity to deliver these services. Community reablement is also highly variable across regions [10] and is unlikely to integrate optimum exercise and nutrition support to target older peoples’ individual needs [11,12], and at a critical time to prevent further deconditioning on discharge into the community. Therefore, development and implementation of exercise and nutrition interventions following acute care is a vital consideration in practice. The effectiveness of these interventions may be influenced by mode of delivery such as remote support options [[13], [14], [15]], and multidisciplinary approaches, particularly those involving volunteer-led initiatives [[16], [17], [18], [19]]. Functional decline due to frailty, and associated challenges in navigating both physical (e.g., traffic) and social environments (e.g., social networks) can create barriers to healthcare access for older adults [20]. Remote exercise and nutrition support options may help to overcome these barriers, increase accessibility to rehabilitation, improve inclusivity and reduce social isolation. Since the coronavirus pandemic, remote provision of healthcare, including frailty management, has become increasingly prevalent [15,21]. Rehabilitation at home is a safe and simple way to improve disability in older people with moderate, but not severe, frailty [22]. A review of telehealth-delivered exercise interventions indicated moderate improvements in older adults’ mobility and strength, and small improvements in balance [15].

Building capacity and capability to motivate and support older adults’ exercise training and nutritional care, requires a multi-agency approach, including contributions from the volunteer sector (i.e., diverse members of the community working for an organisation without getting paid) [22]. Volunteering offers improved psychosocial health for patients and carers, enhances staff capacity within organisations and provides skill development for the volunteers themselves [23,24]. This has led to calls for a strategic integration of volunteering within NHS trusts and wider community healthcare systems, positioning volunteers as an integral part of health-care [25]. NHS volunteer guidance advocates developing inclusive approaches to attract volunteers from diverse backgrounds, enabling NHS trusts to better connect with the communities they serve [25]. For example, research shows the value and acceptability of volunteers from a range of backgrounds, including younger and older volunteers with no clinical experiences to support exercise participation for older people in hospital and community settings, as long as they received support from a central volunteer trainer [26,27].

Emerging evidence highlights the value of volunteer-led physical activity (PA) and nutrition interventions to support older adults’ health after hospital discharge [16,19,26]. A randomised controlled trial found that nutrition-related discussions and strength exercises with older adults performed by trained volunteers, resulted in a 25 % reduction in prevalence of impaired nutritional status, and improvement in frailty [17]. While volunteer-led approaches are not a replacement for professional-led interventions, they are an important adjunct to healthcare. In previous research hospital staff acknowledged challenges with limited staffing and time to promote increased mobility of older people on hospital wards and valued volunteers to support them in that role, in which mobility volunteers were viewed as part of the healthcare team [26]. However, few interventions have explored the feasibility of volunteer-led PA and nutrition interventions delivered remotely (i.e., using online, or telephone-based methods). More insight is needed to explore the acceptability and feasibility of remote volunteer-led multimodal interventions, including development of volunteer support, training and recruitment, and detailed exploration of implementation barriers and facilitators [28]. Furthermore, behavioural change elements have not been considered in previous volunteer-led interventions delivered to older people living with frailty.

A systematic review recommended that home-based health interventions for older people living with frailty need to be underpinned by behaviour change components and highlighted the importance of several behaviour change strategies to improve physical function, including social support, education, and environmental modifications [9]. Hence, a further gap this study addresses is inclusion of volunteer behavioural change training to underpin exercise and nutrition components with behaviour change support. To increase participants’ confidence and autonomy to engage with PA and nutrition, volunteers delivered the intervention using ‘healthy conversation skills’ developed by the NHS initiative of ‘making every contact count’ (MECC) [29]. The MECC approach advocates person-centred conversations and has foundations in self-determination theory, in which health behaviour change, such as improved exercise participation and adherence, are increased and sustained through providing an environment that fosters autonomy (e.g., health behaviours align with personal values), competence (e.g., confidence and skills to engage in health behaviours), and relatedness (e.g., perceptions of personal connection with others) [30,31].

This study aimed to explore the feasibility and acceptability of training volunteers to deliver a remote multimodal intervention, including exercise, behaviour change and nutrition guidance, for older people living with frailty after hospital discharge.

Objectives of this study were to:

1. Develop a training programme for volunteers to support intervention delivery.
2. Assess the feasibility of recruiting, training, and retaining volunteers.
3. Assess the feasibility of recruiting and retaining older adults with frailty to the study.
4. Determine the acceptability of the intervention and explore barriers and facilitators to implementation.

## 2 Methods

### 2.1 Study design

This quasi-experimental mixed-methods feasibility study was conducted at a South England NHS hospital, registered on ClinicalTrials.gov: NCT05384730 (17/05/2022). A feasibility study was chosen to determine whether the remote volunteer-led multimodal intervention was appropriate for further evaluation with a RCT, and to assess whether findings could be shaped to be relevant and sustainable.

### 2.2 Ethics

Ethical approval was received from Health Research Authority (HRA) Wales REC 7 on 30 May 2022 (22/WA/0155). Ethical issues relating to the use of volunteers in delivery of a health intervention to older people with frailty, such as safety and fidelity of intervention components, were addressed through:

1) Volunteer eligibility criteria, including completion of NHS volunteer basic health and safety training and criminal record checks.
2) The development and delivery of a comprehensive volunteer training programme, including competency checks.
3) Leadership and ongoing support from a health practitioner.
4) Establishment of clear volunteer roles, i.e., encouraging and supporting, not prescribing, or changing the intervention.
5) Development and implementation of risk assessments and correct processes to escalate safety concerns.

### 2.3 Volunteer recruitment and training

Volunteers were recruited by hospital voluntary services. Inclusion criteria required volunteers to be ≥16 years, able to provide informed consent and have completed basic clearance checks (e.g., criminal record checks and mandatory training). Individuals unable to safely complete exercises or commit to intervention training and implementation were excluded.

Volunteer training and support, detailed in our protocol paper [32], was conducted by the research team (SJM, LH) and comprised 3 components; 1) seated resistance exercise, including theory and practice 2) Nutrition Wheel training [33], and 3) behaviour change training, via ‘healthy conversation skills’ (HCS) through a self-directed online module. Volunteers were trained to safely facilitate group and one-to-one sessions, with action plans in place to escalate any adverse events to the research team. Once the three training components were completed, the trainer shadowed volunteers during the first month of intervention delivery. Competency was observed and assessed against a framework developed by the research team, which included exercise, nutrition, and behaviour change components (see supplement). Volunteers were encouraged to deliver the intervention independently with continued support (including regular supervision meetings) once their competencies were signed off by the trainer. Additional one-to-one training sessions, emotional and confidence support (e.g., weekly check-ins with the trainer) were available, if necessary, based on fidelity checks and volunteer feedback. Volunteers kept an attendance record during the intervention. The training package was developed and refined through previous research [13,19,26].

### 2.4 Participant recruitment

Older adults on acute medical wards were informed about the study by clinical staff, and if interested, informed consent was obtained by the research team. Inclusion criteria were older adults ≥65 years, able to provide written consent, medically optimised for discharge, with a clinical frailty scale ≥5. Exclusions were inability to safely complete the exercises (per clinician advice), planned discharge to rehabilitation units or care homes, and those receiving end of life care.

### 2.5 Intervention

Trained volunteers delivered a 3-month exercise, nutrition, and behaviour change programme, either online (Zoom), or by telephone, depending on participant preference. Online sessions included 1–8 participants and consisted of real-time group exercise training, followed by group nutrition discussions. Telephone support consisted of one-to-one nutrition support and an exercise review (participants completed exercises in their own time). Volunteers were assigned to days and times to fit their schedule and were encouraged to maintain consistency. Participants receiving telephone support were called weekly, and participants receiving the intervention online were offered support 3 times/week. Participants chose how many online sessions to attend to improve autonomy. Internet-enabled tablets were provided to address digital inequality, and technical support including a home visit to help set up Zoom, and troubleshooting leaflets were available as needed.

Exercises consisted of seated resistance band training for 20–30 min, developed from previous volunteer-led projects [19]. Eight major muscle groups were exercised, and participants were encouraged to progress intensity through increasing repetitions and the resistant grade of the exercise band. Participants were encouraged to complete the exercises 2–3 times per week consistent with physical activity recommendations for older adults [34]. The Nutrition Wheel was used to identify risk of undernutrition [33], and guided participants to appropriate nutritional advice as needed. The Nutrition Wheel is an interactive tool developed from the Patients Association Nutrition Checklist and used to engage individuals in conversation about unintentional weight loss and malnutrition. Based on participants’ answers, volunteers provided suitable nutritional information including educational booklets, recipe ideas, national helplines or, if necessary, signposted participants to their general practitioner (GP)/practice nurse. Consistent with the Nutrition Wheel questions, volunteers also engaged participants in weekly person-centred nutrition topics, such as snacking and fortifying food with additional energy, protein and other nutrients, food shopping, and cooking. Exercise and nutrition discussions were underpinned by HCS, to support positive behaviour change through participant-centred conversations [35]. For a detailed intervention description please refer to our protocol paper [32].

### 2.6 Data collection

#### 2.6.1 Primary outcome measures

Feasibility of implementing the intervention was determined by 1) the number of volunteers recruited, trained, and retained, 2) participant recruitment, retention, and adherence to the intervention, and 3) recorded adverse events. Adherence to the intervention was assessed objectively by the proportion of sessions participants attended, which was recorded by volunteers using session registers.

Acceptability was explored via semi-structured interviews with older adults, carers, and volunteers conducted by SJM and LH in-person or over the telephone. Interviews were audio-recorded and guided by Normalisation Process Theory (NPT) to explore social implementation processes, barriers and facilitators [36].

#### 2.6.2 Secondary outcome measures

Secondary outcome measures included:

1) Self-reported PA measured using the physical activity scale for the elderly (PASE) [37].
2) Device-based PA assessed using wrist-worn accelerometers (GENEActiv, Activinsights, Kimbolton, Cambridge, UK) were used to examine total PA (TPA), light PA (LPA), and moderate-vigorous PA (MVPA) [38]. GENEActiv accelerometers measured triaxial movement acceleration in gravity (g) units (1 g = 9.81 m/s2) at a frequency of 100 Hz continuously over a period of 7 days.
3) Activities of daily living measured using the modified Barthel Index [39].
4) Appetite measured using the Simplified Nutritional Appetite Questionnaire (SNAQ) [40].
5) Well-being assessed using the Warwick-Edinburgh Well-Being Scale (WEMWBS) [41].
6) Anxiety and depression symptoms measured using the Hospital Anxiety and Depression Scale (HADS) [42].
7) Quality of life (QOL) measured using the EuroQol-5 Dimensions-5 Levels (EQ-5D-5 L) questionnaire [43].
8) Self-efficacy in managing chronic disease measured using the 6-item Lorig scale [44].

A description of each outcome measure is detailed in our protocol paper [32]. Secondary outcomes were assessed at baseline (0–7 weeks post-discharge), post-intervention, and 3-months follow-up.

### 2.7 Analysis

Statistical analysis was conducted using the statistical software SPSS (V.29). Feasibility measures were analysed using descriptive statistics, including median (interquartile range [IQR]); mean (standard deviation [SD]); and number (%). Secondary outcome measures were assessed for normality and described using parametric or non-parametric statistics accordingly. Baseline outcomes were compared with measurement post-intervention and at 3-month follow-up using repeated measures analysis of variance (ANOVA) for normally distributed data, and Friedmans tests for non-normally distributed data. Statistical significance was considered when *p* < 0.05, and adjusted p-values were applied for pairwise comparisons. A basic cost-analysis of the training programme was performed.

### 2.8 Accelerometer data processing

GENEActiv data were processed in R (R Core Team, Vienna, Austria) using the open-source GGIR software package (http://cran.r-project.org). Previously validated acceleration threshold values (in older adults) were used to quantify the time (minutes.day^−1^) spent on average in each intensity category: TPA, LPA, MVPA, and sedentary time (sedentary to LPA = 255 g min^−1^; LPA to MVPA 588 g min^−1^, for 60 s epochs) [38]. Accelerometer non-wear time was identified using the standard deviation and the value range of each accelerometer axis [45]. Days where accelerometer wear-time was <16 h and, or participants with <4 days wear-time were excluded from analyses.

### 2.9 Analysis of intervention acceptability

Interviews were transcribed verbatim and analysed using reflexive thematic analysis (RTA) [46]. RTA offered a nuanced, detailed understanding of the implementation barriers and facilitators, capturing older adults’ complex meanings assigned to their health and recovery after a hospital stay, and the multicomponent intervention supported by volunteers. RTA was performed by SJM, who is a white British female researcher with a background working as an exercise instructor. Initially SJM aimed to analyse the data deductively using concepts and categories generated by NPT [36]. However, the familiarisation process revealed complex meanings and narratives beyond purely using NPT in a deductive way. Therefore, to situate the analysis close to participants’ lived experiences SJM inductively coded the data, capturing both semantic and latent meanings behind the data, and then used NPT to facilitate understanding of these meanings within an implementation framework. From a relativist (i.e., knowledge is relative to differences in perception) and constructivist (i.e., knowledge is constructed and subjective) position SJM coded and grouped the data into themes working in an iterative way through the six phases of RTA, described in detail elsewhere [46]. NVivo software (V.12) was used to organise the data during RTA.

## 3 Results

### 3.1 Feasibility of delivering the volunteer training programme

Five volunteers were recruited and 3 were retained (60 %) at the end of the study (Table 1). Most volunteers did not have previous volunteering experience (60 %), and no experience delivering exercise (80 %), or nutrition support (100 %). Volunteers participated for 16–38 weeks (mean 26.20 ± 7.60). Two participants withdrew from volunteering due to college commitments.

**Table 1:** Characteristics of volunteers and participants signed up to the Frail2Fit intervention.

Characteristics	Participants (*n* = 26)	Volunteers (*n* = 5)
Age^a^ (years)	80.3 ± 7.0	16.40 ± 0.49
	67-94 (range)	16-17 (range)
Gender		
Male	11 (42.3%)	2 (40%)
Female	15 (57.7%)	3 (60%)
Ethnicity		
White British	25 (96%)	2 (40%)
White South African	1 (4%)	0 (0%)
Asian British	0 (0%)	1 (20%)
Chinese	0 (0%)	1 (20%)
Polish	0 (0%)	1 (20%)
Marital Status		
Divorced	7 (26.9%)	0 (0%)
Married	11 (42.3%)	0 (0%)
Widowed	7 (26.9%)	0 (0%)
Single	1 (3.8%)	5 (100%)
Care		
No Care	11 (42.3%)	
Formal Provision	13 (50.0%)	
Informal Provision	2 (7.7%)	
Residence		
Private home living alone	6 (23.1%)	
Private home living with family	12 (46.2%)	
Sheltered accommodation	8 (30.8%)	
MMSE^b^	28 (24.8-29.0)	
	21-30 (range)	
Charlson Comorbidity Index^b^	4 (4.0-5.3)	
Clinical Frailty Scale^b^	6 (5-6)	
	5-7 (range)	
No. of Medications^b^	9 (6.0-11.3)	
	0-14 (range)	
0-5	5 (19.2%)	
6-10	13 (50.0%)	
>10	8 (30.8%)	
Length of hospital stay (days)^b^	15 (8.8-22.5)	
	2-44 (range)	
Smoking status		
Never	11 (42.3%)	
Ex	13 (50.0%)	
Current	2 (7.7%)	
Pack years^b^	4.0 (0.0-23.5)	
	0-55 (range)	
Alcohol consumption^b^	0 (0-6.0)	
	0-14 (range)	
BMI (kg·m^2^)^b^	25.5 (20.7-28.0)	
Underweight (<18.5)	2 (8%)	
Healthy weight (18.5-24.9)	11 (42%)	
Overweight (25-29.9)	8 (31%)	
Obese (30-39.9)	4 (15%)	
Severe obesity (>39.9)	1 (4%)	
Reason for hospital admission		
Fall	11 (42%)	
Infection	4 (15%)	
Acute Kidney Injury	2 (7%)	
MSK causes	2 (8%)	
Metastatic liver lesion	1 (4%)	
Hyponatraemia & delirium	1 (4%)	
Abdominal pain	1 (4%)	
Lymphoedema	1 (4%)	
Aortic dissection	1 (4%)	
Giant cell arteritis	1 (4%)	
Peripheral oedema	1 (4%)	

a mean ± standard deviation.

b median and interquartile range; MMSE, mini-mental state examination; BMI, body mass index.

Each volunteer completed a median of 5.5 h (IQR 4.75–7.0) of training. The cost of training a volunteer was estimated to be £160.12. The trainer (SJM) shadowed each volunteer for 1–2 sessions before their competencies were signed off. Once volunteers completed their competencies, they were encouraged to lead the support sessions from the hospital office where a member of the research team was on-hand if required.

### 3.2 Feasibility of delivering the frail2fit intervention

**Fig. 1:**
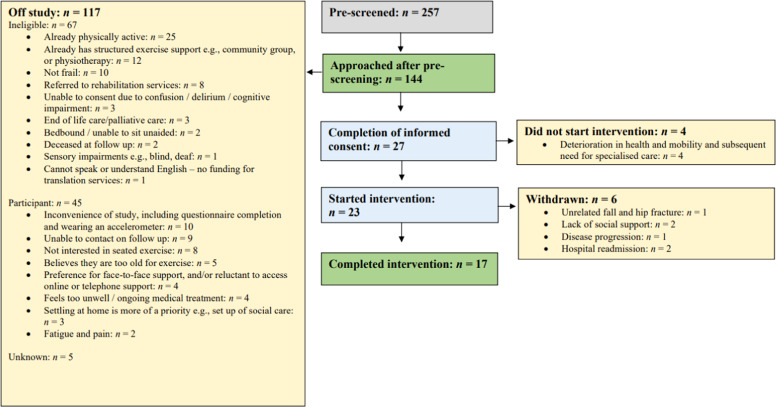
Frail2Fit screening and recruitment flow diagram.

Participant characteristics are presented in Table 1. Twenty-seven participants signed up to the intervention, and 17 completed (Fig. 1). Eight participants completed the intervention online, and 9 opted for telephone support. Overall, volunteers delivered 45 online sessions and 90 telephone calls January–October 2023. Volunteers completed 2–14 online sessions each (median 11 [IQR 6.5–13.5]), and 10–36 support calls (median 13 [IQR 10–28]).

### 3.3 Adherence

Over the 3-month intervention participants in the online group completed 4–15 sessions each (mean 10 ± 3.07), and participants in the telephone group received 6–9 calls (mean 7.11 ± 1.10). Of the online sessions offered, participants had a 19–92 % (median 75 % [IQR 38–92]) attendance rate, and of the telephone calls arranged, 67–100 % of the calls were completed (median 80 % [IQR 68.5–94.5]).

### 3.4 Nutrition wheel

Eighteen participants completed the nutrition wheel and 9 (50 %) were considered at risk of malnutrition. Of those at risk of malnutrition all were offered resources in the post, and 5 accepted the nutritional advice.

### 3.5 Secondary outcomes

Self-reported TPA (PASE), QOL (EuroQol Index), and appetite improved significantly post-intervention with a sustained improvement in TPA and appetite at 3-month follow-up (Table 2). There was a non-significant improvement in functional ability (Barthel), self-efficacy, QOL (VAS), and well-being post-intervention. There were no significant differences in anxiety or depression over time (Table 2).

**Table 2:** Secondary outcome data.

**Outcomes**	Baseline (*n* = 17)	Post-intervention (*n* = 17)	3-month follow-up (*n* = 17)	Significance (*P* < .05*)	F-Statistic	Post-hoc pairwise comparisons		
					χ^2^-Statistic	B-P	B-F	P-F
PASE Score^a^	32.8 ± 35.2	50.8 ± 31.1	46.2 ± 39.8	*p* = .01*	F_(2, 32)_ = 5.26	*p* = .006*	*p* = .04*	*p* = .44
Modified Barthel Index^b^	18.0 (14.0–20.0)	19.0 (17.5–20.0)	20.0 (16.5–20.0)	*p* = .14	χ^2^(2) = 5.7			
	6.0–20.0 (range)	7.0–20.0 (range)	4.0–20.0 (range)					
EuroQol VAS^a^	58.2 ± 24.2	65.6 ± 22.4	55.8 ± 22.9	*p* = .28	F_(2, 32)_ = 1.3			
EuroQol Index^b^	0.683 (0.489–0.708)	0.733 (0.570–0.803)	0.720 (0.456–0.813)	*p* = .04*	χ^2^(2) = 6.48	*p* = .04*	*p* = .31	*p* = 1.0
	−0.098–0.840 (range)	0.365–0.950 (range)	0.132–0.942 (range)					
SNAQ^a^	14.2 ± 3.3	15.4 ± 2.3	15.6 ± 2.0	*p* = .01*	F_(2, 32)_= 5.05	*p* = 0.03*	*p* = 0.01*	*p* = 0.56
SNAQ <14	6.0–18.0 (range)	10.0–18.0 (range)	11.0–18.0 (range)					
	*n* = 7 *(*41 %)	*n* = 3 (18 %)	*n* = 2 (12 %)	*p* = .02*	χ^2^(2) = 8.4			
WEMWBS^a^	49.5 ± 8.2	50.2 ± 9.3	51.4 ± 9.6	*p* = .50	F_(2, 32)_ = 0.71			
	32.0–61.0 (range)	30.0–61.0 (range)	30.0–62.0 (range)					
Self-efficacy^a^	37.4 ± 9.2	41.8 ± 6.5	40.9 ± 10.2	*p* = .13	F_(2, 32)_ = 1.9			
	19.0–51.0 (range)	30.0–51.0 (range)	22.0–55.0 (range)					
HADS^b^								
Anxiety	4.0 (2.0–6.5)	4.0 (2.0–6.5)	4.0 (2.5–6.0)	*p* = *.96*	χ^2^(2) = 0.30			
Depression	0.0–11.0 (range)	0.0–10.0 (range)	0.0–8.0 (range)					
	6.0 (3.5–8.0)	3.0 (1.0–5.0)	4.0 (3.0–7.0)	*p* = .03*	χ^2^(2) = 6.89	*p* = .10	*p* = 1.0	*p* = .08
	0.0–14.0 (range)	1.0–11.0 (range)	1.0–16.0 (range)					
Device-based (GENEActiv) 24-h movement behaviour								
Compliance^b^	Baseline	Post-intervention	3-month follow-up (*n* = 16)	Significance				
	(*n* = 16)	(*n* = 16)	6 (6–6)	(*P* < 0.05)				
Total days worn	6 (6–6)	6 (6–6)	1.36 (0.37–2.67)					
Non wear ( % per day)	0.79 (0.34–3.55)	0.87 (0.18–9.73)						
Physical activity (mins∙day^-1^)								
Inactive^a^	751.42 ± 51.86	744.39 ± 43.84	731.01 ± 52.69	*p* = .18	F_(2, 30)_ = 1.84			
Light^a^	29.95 ± 21.07	38.88 ± 24.98	33.37 ± 24.67	*p* = .10	F_(1.48, 22.18)_ = 2.79			
Moderate-Vigorous^a^	18.72 ± 17.25	23.08 ± 18.87	21.02 ± 18.12	*p* = .30	F_(1.23, 18.37)_ = 1.20			
Total^a^	48.67 ± 37.83	61.96 ± 43.24	54.39 ± 42.16	*p* = .16	F_(1.37, 20.56)_ = 2.11			

PASE, physical activity scale for the elderly; VAS, visual analogue scale; SNAQ, simplified nutritional appetite questionnaire; WEMWBS, Warwick-Edinburgh mental well-being scale; HADS, hospital anxiety and depression scale; LPA, light physical activity; MVPA, moderate-vigorous intensity physical activity; B, baseline; P, post-intervention; F, 3-month follow-up. 18 % of baseline data was collected in hospital either on the participant’s date of discharge, or 1 day before discharge, and 82 % of baseline data was collected out of hospital 3.9 ± 1.4 weeks post hospital discharge.

a Mean ± SD.

b Median (Interquartile Range).

Multiple imputation was used to handle missing GENEActiv data (8 % of cases), in which 5 imputed datasets were aggregated into a final pooled dataset for analysis. There was a non-significant improvement in inactivity, LPA, MVPA, and TPA post-intervention (Table 2).

### 3.6 Adverse events

One adverse event was reported in which a participant had shoulder pain after strength training. Volunteers escalated the adverse event to the trainer (SJM), who contacted the participant to modify their exercise program.

### 3.7 Acceptability of the frail2fit intervention

**Fig. 2:**
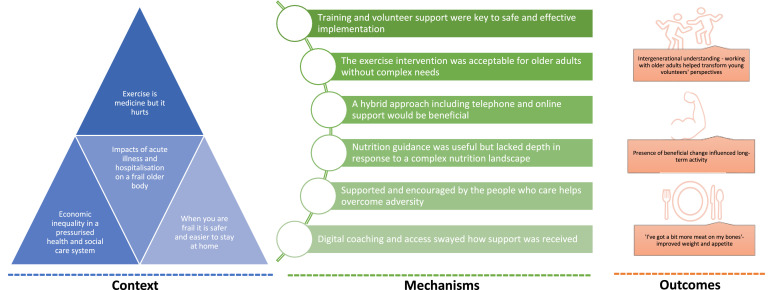
The main themes and subthemes showcasing the acceptability of the Frail2Fit intervention.

Sixteen older adults (aged 67–92 years; 10 female), one female carer, and five volunteers (aged 16–17 years; 3 female) were interviewed. Thirteen sub-themes have been organised under three higher order themes influenced by NPT [36]: implementation contexts, mechanisms, and outcomes (Fig. 2). Table 3 illustrates themes with data extracts edited to remove superfluous material.

**Table 3:** The main themes and subthemes influencing the acceptability of the intervention have been displayed with supporting quotations, detailed with participants’ contextual information (participants have been given pseudonyms to protect anonymity).

**Main theme: Implementation context**		
**Subtheme L2**	**Subtheme L1**	**Quote examples**
Impacts of acute illness and hospitalisation on a frail older body		Stephanie, the wife, and main carer of George (aged 85, CFS 7) described her frustration with the lack of encouragement George received to move independently: ‘During his time in hospital he was scarcely ever out of bed. And if he was out of bed, he was on the Sara Stedy, so he wasn’t walking. And I found it very difficult - everybody’s in pyjamas…. And I s’pose that’s safer for them (staff), because nobody can accuse them of neglect if somebody falls.’ Alice (aged 77, CFS 5) reported a lack of encouragement from staff for patients to use the toilet independently: ‘I think they looked a bit surprised when I asked if I could go to the loo after I’d been stuck in bed for ages. They sent someone to escort me to the loo and back. I was a bit concerned that they didn’t encourage you to get up and go to the loo… It’s much easier for them to stick an incontinence pad on you than to get up and take you to the loo and wait for you outside, and then walk you back to your bed.’ Carley (aged 72, CFS 6) showed the impact of illness on her wellbeing and confidence: ‘It just feels as though my whole body seems to be playing up a bit and I’m not that old. And so it’s had an effect on my wellbeing, not just my physical, but psychological wellbeing as well… Yeah, it takes a bit of confidence to move around now too.’ Beryl (aged 67, CFS 6) described how simple every day movements were an effort: ‘Just to go out into the kitchen and lift the kettle up to make a cup of tea was so difficult. And I was so shocked at how weak I felt when I came home from hospital’.
When you are frail it is safer and easier to stay at home	Accessibility is challenging	Edith (aged 78, CFS 5) showcased her fear mobilising in an unsafe environment and liked the idea of remote support: ‘I’ve got a walker which I’m not 100 % happy, or comfortable using, because the pavements are so rickety with all the digging up that they’ve done over the year. And another thing is the pavements aren’t very wide. We have such a lot of heavy traffic, transporters, and skip lorries; big container lorries come thundering down, and if you’re a bit unsteady on your legs it’s a bit daunting really. Especially when they go by, there’s this great rush of air that’s left…. I’m frightened of falling over which doesn’t help…. I think it’s good you can do these zoom meetings - I thought it was ideal. I mean, it’s easier than going to a Church Hall and doing exercises in a class – which I did do before I had my hip (operation).’
	Internalising negative stereotypes and societal views	Karen (aged 76, CFS 7) highlighted experiencing disability stigma: ‘It’s difficult when somebody doesn’t understand how you are - I am not stupid! Because you sort of stop in the middle of sentences, and forget words, and things like that, doesn’t mean to say you’re stupid… And I hate it when people assume that you’re stupid, that you’ve got no dignity… It’s more to do with being in a wheelchair I think - how they perceive you.’ Alice demonstrated how feeling self-conscious of her ageing body and the way her ageing body looked and performed led her to give up yoga: ‘I did Yoga for 20 years; I’ve given it up, because I got so puffy at the beginning of the year. I didn’t want to be the old person in the corner that everyone was looking at. And I think that’s the other thing, you got to feel comfortable in a group. And you’ve got to choose the group where people are going to be like you. It’s no good doing a class full of fit 35 year olds.’
Economic inequality in a pressurised health and social care system		Daniel (aged 68, CFS 6) felt lonely, and neglected by health and social care: ‘Nothing at all. He (consultant) said do what you want – have a cigarette, have a glass of wine… that’s the only response I got…… I see three carers, they don’t talk, they just come in and go. I don’t have anyone I can have a conversation with. So, I’m stuck in front of the television all the time, all day…… They (carers) cook my breakfast, lunch, and dinner and they make my bed, and that’s about it really. But a couple of weeks ago, I got food poisoning and I found out what it was; it was a carer giving me out of date food! I was on the toilet for three days running!’ After intermediate care finished, Sophia (aged 88, CFS 6) was able to afford private care with carers who had the capacity to help with her meaningful activities, including support with intervention exercises and walking: ‘It’s only 2 weeks (intermediate care). So, I thought, well, I don’t really want these (carers) anyway, cos they don’t do what I want them to do; no, a bit lazy; can’t see for themselves….. These other people I pay, they work for themselves; they’re highly trained; they’ve been in hospitals and working agencies, but they do it for themselves now…. We do the exercises every day, and more so sometimes than it says… I’m doing it all with my carers. We did more than 8 (reps) - one of the carers was so vigorous. And we’ve got these bands of course. My yellow one’s up there. So, I’m egged on by them, they don’t let me slack off… if you’re with someone, they encourage you, ‘we’ll do our exercises now’. So, you go and do it…’
Exercise is medicine but it hurts		Alice illustrated the ‘dilemma’ between beliefs that exercise is beneficial for health and the difficulty of exercising with frailty: ‘It’s a progressive disease which has no cure; nothing is going to alleviate it other than exercise, which will stabilise it… if physical exercise were a pill, it would be the most successful drug ever invented…… The problem is when you’re old, and tired, and you can’t breathe, it is difficult to motivate yourself to do it. Whereas, when you’re younger, and you don’t have any of these problems, it’s hard even then, but when I was younger, I used to get up and go to the gym for an hour before I went into the office or take the dog for a 4-or-5 mile walk. I never used to think about it; it just used to be part of my routine. But, I think, when you get older, and you get medical problems, it becomes much more difficult, and yet, it’s a trap - you know that you ought to do it, but then your body says, ‘ah, can I just sit in this chair and watch TV’. It's a dilemma, so anything that makes you do it has got to be good.’
**Main theme: Implementation mechanisms**		
**Subtheme L2**	**Subtheme L1**	**Quote examples**
Training and volunteer support was key to safe and effective implementation	‘Whenever I didn’t know what to say, she’d always be there’ – inexperienced volunteers needed practical and emotional support	Josh (volunteer) alluded to the support he received from the trainer: ‘She told me a lot about what I should be doing. And she was very patient with me. She was always very encouraging; always very positive. I felt like that’s a very important thing. You want to encourage people; you want to make sure they stick with it to the end. Whenever I didn’t know what to say she always stepped in and helped me. And it was very good to see someone there always to support me, teach me what to do, and what to say…… There were a couple of people that did not get much better. It was quite difficult hearing their stories. Some of them suffered terrible falls, and they had to be re-admitted to hospital and they had this pessimistic view on their lives. I didn’t really know what to say to them. I tried to be sympathetic…. really there’s nothing I could do… gradually I did get better dealing with situations like these. Whenever I didn’t know what I was going to say, she’d (trainer) always be there. And she’d always take over the phone call. She’d always teach me how; different things to say in different kinds of situations. That was quite helpful.’
	Training structure and resources shaped volunteer expectations and prepared them for their role	Aidan (volunteer) approved of the training content and structure: ‘I enjoyed the training; it was straightforward; pretty easy to learn; to understand… It was everything I needed to know… I was trained in the exercises first, so I had a bit more time to learn each exercise, and when I could apply it, I could apply it well. With the nutrition support, there was less to learn so I picked it up quickly. And then, I delivered the nutrition support as well as I would have.. I actually felt it was very well set up. And you have the online site where you can track your hours. And the booklet which helped you understand everything about the exercise. I don’t think anything was missed.’ Emily (volunteer) found role play useful: ‘I liked with the exercises we were able to go through them; that you showed us how to do it. I thought that was extremely helpful. And that we had a go. Because, I feel doing them in front of people first, if you’ve never done such a thing before, is quite stressful. And it’s a bit awkward. So, I think the fact that we got to do it during our training was good because showed us that it’s not actually that scary. And it got us into that mind set.’
The exercise intervention was acceptable for older adults without complex needs		Thomas (aged 83, CFS 6) indicated the value of progressing resistance training: ‘The bands are very good aren’t they? Cos they give that little bit more… I can do all these things (exercises), quite easily now. So, I prefer to use the stiff one (resistance band). I use that all the time now.’ Shirley (aged 78, CFS 5) found the intervention helped build her confidence to exercise: ‘It (seated exercise) sounded less demanding. And I felt that may be an incentive to keep me doing it, which has proved to be the case. I have found myself doing it on non-zooming days a lot more that I thought I would.’ Carley would have liked to progress to standing exercise: ‘I was interested that we didn’t do any standing up exercises; it was all seated. And I wondered what the reason for that was. I know that the Physiotherapist that I saw at Southampton, she was encouraging me to march on the spot, and try going upstairs, leading with both legs after a while. And standing at a table or counter, and moving my legs to the side. I tried to do that a bit as well. But in the group exercises, it was all seated, wasn’t it?’
A hybrid approach including telephone and online support would be beneficial		Jenny (volunteer) spoke about online nutrition support: ‘The thing with being online, I think it’s quite a sensitive topic. So, I think people would want to talk about it on their own. If you were to do it in a group, as well, you have to go a bit faster to get through everyone. And, people might not elaborate as much, and, again, people might not want to share because of other people there. So, I think it was good for generalising, and certainly for the wheel, for the … questions really, but for a general chat about nutrition I think it was a bit harder to do it in a group. Just because there were too many people I think.’ Aidan (volunteer) explained the advantage of exercising online: ‘The exercise session online, that was definitely helpful because you could see, the patients could see how you were doing the exercises. So, they could imitate it, which you wouldn’t be able to do on a telephone call. The patients who have the telephone support, one question I did get asked was, about halfway through the 3-month stint, they did wonder if they were doing the exercises right, cos you can’t visualise the exercises on the phone calls.’ Teresa (volunteer) saw benefits and disadvantages from both modalities and suggested a hybrid approach would work better: ‘I feel a blend between zoom and telephone support. So, calling the people that do zoom every couple of weeks, or something to get an in-depth update of where they’re at would help a bit more to know exactly how each participant is doing.’
Nutrition guidance was useful but lacked depth in response to a complex nutrition landscape		Jenny (volunteer) found the nutrition wheel a good conversation starter: ‘I think it was helpful for us starting it (nutrition chat) because the questions…. it gives them something to talk about. And I think that was good because they were encouraged to talk more about their situation.’ Sophia used the nutrition wheel advise to fortify her meals: ‘I make a salad, and I have smoked salmon or ham. I always have a boiled egg; I have a lot more eggs now. And I have cheese, so keep building it up. Like when you’re eating the little new potatoes, put butter on, or spread on, so there’s lots of things like that… I am putting on weight. Quite slow, but I am.’ Joyce (aged 86, CFS 5) showed the influence of traditional diets on her perception of nutrition advise in the programme: ‘I could have contradicted because a lot of things that I learnt I’ve had to relearn. Because, over the years, they’ve changed. Something that we put into diets in 1950, are now taboo. And those things have never left my mind. And I did feel that, because the rest of the group of us were of the same age era, that they’d probably perhaps didn’t know, or understand, the up-to-date. It’s a funny thing to say, ‘they say now, no salt; people shouldn’t have salt in things’. Well, I was brought up, in our era, I was brought up with salt being put into vegetables to cook. And I still do put salt in, because if I don’t I get cramps.’
Supported and encouraged by the people who care helps overcome adversity	Volunteers were caring and joyful	Samuel (aged 70, CFS 5) showed the non-judgmental support he received from the volunteers: ‘What was very good was they didn’t push it too much. When I said I exercise 3 times a week, they didn’t push that and say, ‘oh, you should be doing this; you should be doing that’ - they didn’t push anything… They just encouraged you, which was good…. They were polite, finding out what I’d been doing, and what I hadn’t been doing I s’pose but not criticising me for not doing it… They’re concerned about you, getting people back to normal, aint they?’ Shirley highlighted the transfer of positive emotions from the volunteers: ‘Lovely. Cheerful, Emily, that little smiley face every time was just lovely. And it just cheered up the day, it really did. Jenny, a little more serious, but still very confident; very friendly, and cheerful.’ Joyce indicated that volunteers built up her confidence to exercise: ‘I’d like to think that they’d go on and do something because they’d give anybody confidence…. They were giving you confidence to do the exercises.’ Emily (volunteer) showcased how she gave participants emotional support: ‘I’m a very sensitive person, so it did make me quite upset inside for that person. But I was just happy that I could be there, and just listen to her - kind of help by just listening. It’s difficult in that situation because you don’t want to say something that’s not true like, ‘it will get better’, because you never know, it could get worse. But you want to be there for that person as much as you can. I didn’t know her very well, I didn’t know what to say, but I think just listening and kind of being very supportive, so she knew that I was emotionally with her. And I wasn’t judging her or anything. I think that was important to her. I think she really needed that as well. So, I was glad I could give that, and it was hard, but I think it wasn’t something I couldn’t handle. I think me handling it helped her as well which is all you can ask for really.’
	Online group support was like a virtual cuddle	Shirley felt a sense of relatedness in the group and described how she helped to reassure others: ‘I enjoyed doing the one online. It was interesting talking to people to see what other people were feeling and how they were all doing…. You sort of get that feeling that you’re part of something… it was fun. Strangely enough, I would find myself looking forward to the next one….. I found her (participant) progress was very slow, but she seemed to want the praise from me because I’d been through it already, in having my hips replaced. I said, ‘oh, that’s really good’, I could see her smile, and I felt it was encouraging her to keep going. Because, in the beginning, she was in a lot of pain, and she’d been through a lot. And she needed that little booster, that little cuddle even if it was over the air, through the zoom. I built up quite a friendship.’ Emily (volunteer) indicated the group was a positive vicarious experience for older adults experiencing similar problems and helped reduce loneliness: ‘I think being around the same sorts of people helped her. Because she did get better - emotionally, and physically. I think the others inspired her in a sense that, she saw that, ‘aw, they’re doing it; they’re going online;’ and they were determined as much as she was. And I think that gave her a bit of support, because there’s a limit to how much a 17 year old can give support about someone’s physical condition, in the older aged, if that makes sense. I’m very well, and fit, and everything, and I don’t have any conditions. So, I think surrounding herself with people, similar age; similar problems; is very good, cos it allows her to not feel as alone…. It’s the little talks as well, that benefitted them. Especially if someone lives alone, even around a stranger or a volunteer asking them, how was your day? I think that can have a positive impact… That feeling someone is interested in them; and someone actually cares about their day. I loved, during these little chats, I saw a lot of smiles.’
	Surrounded by friends and family’s love was vital to recovery	Stephanie was the main carer for her husband, giving George endless encouragement and love in the hope that his health would improve: ‘I tried to fit them (exercise) in wherever we could. And sometimes he was keen, and sometimes he wasn’t. So, on the whole, we were managing exercises 4 times a week. And we gave ourselves a rest on Sunday.’ Karen’s husband ensured she ate properly: ‘I eat better because my husband cooks. I never was one to eat well, because I just didn’t eat. But he cooks for me; makes sure I have a meal every day. I wouldn’t bother.’ Beryl highlighted the support she received from her sisters: ‘My sisters, oh boy, I don’t know what I would have done without them?! My sisters were going shopping for me. And she works full-time, bless her heart, and she’s still doing this for me and my other sister. I would sit here Thursday evenings and write a list. And then, Friday mornings, she would phone me, and I would relay down the phone what I needed, and she’d write the list her end. And then, bless her heart, she would go shopping. She would deliver it, and she’d pack it away; and we’d have a hug, and I’d say, ‘thank you’, and off she would go. And that’s how I did it, so I had food. I couldn’t go out; I wasn’t well enough. There was no way; I couldn’t even walk; the idea of just walking from my front door to the gates, it was like, ‘I can’t do it’. it would have been like walking a mile.’
Digital coaching and access swayed how support was received		Samuel opted for telephone support, indicating his struggles with using digital software due to his perceptions of being too old and his fear of artificial intelligence (AI): ‘The computer itself I’m hopeless. So, AI won’t affect me… I don’t use it. It’s a younger generation thing, init?… Cos I’m still living in the last century, that’s why. I’m hopeless online, I don’t do things online much.’ Mark (aged 85, CFS 5) described his fear of being scammed through using unfamiliar technology and the help he received from his wife: ‘I’m 85, how the hell can I talk to a computer?! ….. And then, this business about people taking all your money. If anything comes up, and I’ve got to pay, that’s it. I just delete it…… My wife would say, ‘no, Mark, press this button; press that button’. I haven’t got a clue. She done all of it. If it weren’t for my wife I wouldn’t be doing it.’ Carley showed her previous digital knowledge and experiences helped her access online support sessions: ‘I guess I use zoom quite a bit, so, I mean, in that sense there was no problem. I didn’t have to be trained about it to join.’ Beryl received digital support from the research team: ‘Thanks to you, you helped me with that by bringing me a tablet to make use of until the course finished… If you remember the couple of times I phoned I perhaps got a little bit, you know, not terribly good with things, computers and so on, cos I don’t possess one of my own. But I was encouraged, and people was very helpful; helping me to get it right so that I could go online and join in.’
**Implementation outcomes**		
**Subtheme L2**	**Quote examples**	
Intergenerational understanding – working with older adults helped transform young volunteers’ perspectives	Participants’ illness stories transformed Josh’s (volunteer) perspectives on some of the struggles older adults with frailty endure: ‘It was definitely very difficult, but I feel it was important to hear about these sorts of things - to really hear about people’s perspectives. It’s not something we hear about every day, so, it’s important to acknowledge that there are people like this in this world.’ Emily (volunteer) explained how she helped change older peoples’ negative ageing stereotypes into a more positive outlook: ‘I had someone tell me that, ‘I’m 80, I’ve got nothing to look forward to anymore’. That really stuck with me, I thought, ‘that shouldn’t be the case’, because an 80 year old could still live for 30 years; there’s still so many good things you can do when you’re older. If you stay fit and healthy, then you can do more of those things. And you cannot lose that sense of life, and happiness in life, and that motivation, which I think is important because age doesn’t matter. I mean, obviously, as you grow older, you’re less energised, but there’s still so many things you can do. If you lose all that hope, then you don’t get to experience all that stuff. So, I think it’s important to give that reassurance, and motivation - it’s important to raise that thought with older people.’ Jenny (volunteer) found a renewed sense of confidence and altruism through volunteering: ‘I want to be a Dentist in the future and I think this really helped with my communication skills with older people, and it brought me out my comfort zone. I think that’ll really benefit me in the future…. It really boosted my confidence talking to someone on the phone, I used to be really nervous…. Helping others makes you feel fulfilled in what you’re doing, and I think that’s the biggest takeaway, seeing me help other people; that’s really benefitted me.’	
Presence of beneficial change influenced long-term activity	Shirley experienced exercise benefits, motivating adherence: ‘I have 3 trigger fingers between the two hands. And since doing the hand exercises, they are triggering a lot less than they ever have before… I have found myself doing it on non-zooming days a lot more than I thought I would. Cos, my family all went, ‘oh, yeah?!’ - I think I’ve surprised most of them… I do need to keep going with the band… If I’ve gone through the programme with the bands, I must admit afterwards, I feel quite perky, awake, and alert.’ Thomas was put off exercising from a lack of perceived progress: ‘I hardly progressed at all… The net result, after all these weeks, I don’t feel much better than when I started, which is a disappointment. I thought they (exercises) would (help me), because exercises are good, and I expected them to bring me on quite quickly. But it hasn’t worked out that way… I feel bad if I don’t do them - I’ll regress. But I’m not improving to what I expected. I don’t want to regress, going back rather than forward. But the trouble is, it doesn’t seem to be improving. If I was (improving) I would readily be going through them (exercises) again.’	
‘I’ve got a bit more meat on my bones’ – improved weight and appetite	Margaret (aged 75, CFS 5) reported improved weight after returning home: ‘I put it on very quickly. Obviously, the drinks (oral nutritional supplements) were helping, and perhaps I was eating a bit better when I come home, cos I wasn’t all that keen on the hospital food.’ Beryl had reduced appetite after discharge from hospital and indicated exercise as a way to improve hunger: ‘I would force myself to eat; actually, make myself eat; ‘I’ve got to eat this!’ And it would be a real struggle to chew it and swallow it. But I thought, ‘I’ve got to do this’, because I’m gonna waste away, cos I knew I’d lost weight. I was looking skinny - all my clothes looked like a sack……. I felt good after I’d finished the (exercise) session on the Monday. I used to think it won’t be long until lunchtime, so I’d be feeling even peckish…. My clothes size is 10, and they were just hanging on me. I can still fit my hand in there (trouser leg), but it’s not as bad; I’ve got a bit more meat on my bones…… I’m now able to go out and choose what I want to buy myself, seeing it on the shelf and in the freezer - I think, ‘oh, I fancy that!’’	

### 3.8 Contexts

#### 3.8.1 Main theme: impacts of acute illness and hospitalisation on a frail older body

Participants had reduced strength and function at hospital discharge, resulting in diminished independence and need for formal and informal care, which impacted confidence and wellbeing. Participants reported a lack of PA on the wards, which had a significant impact on functioning. Staff perceptions of older adults’ ability to mobilise safely in hospital influenced dis/encouragement of patients’ independent activity. Staff didn’t have the capacity to support patient’s mobility, subsequently it was easier if patients stayed close to their beds, for example, staff dissuaded independent toilet use. Isolation due to infection control reasons and poor availability of walking aids were also reasons for participant’s reduced activity in hospital.

#### 3.8.2 Main theme: when you are frail it is safer and easier to stay at home

Physical restrictions and decreased confidence after acute illness caused challenges for older adults to leave their homes. The remote support provided was an appropriate format to reduce social isolation and improve intervention inclusivity.

Subthemes:

1. *Accessibility is challenging*

Most participants felt unsafe mobilising out of their homes due to physical restrictions, and reduced confidence. For example, alterations in senses such as hearing and eyesight, prompted older adults to give up driving. Participants illustrated a lack of confidence to mobilise out of their homes due to poor pavement quality and traffic, and a fear of falling. Therefore, participants were appreciative of remote support.

2. *Internalising negative stereotypes and societal views*

Older adults with frailty were more likely to stay home and were careful where they exercised to avoid judgment from others, built from internalising negative ageing stereotypes and disability stigma. Feeling their bodies were being negatively judged led some older adults to avoid intergenerational exercise spaces, and individuals with debilitating long-term health conditions were frustrated with society’s lack of symptom understanding, indicating instances of disability stigma.

#### 3.8.3 Main theme: economic inequality in a pressurised health and social care system

Older adults with frailty who were isolated and receiving inadequate social support struggled to participate in the intervention. Insufficient personal care was described as a key barrier to intervention engagement, in which participants concentrated their limited energy on receiving care for their basic needs. For example, a female participant with Parkinson’s Disease explained carers put her to bed for the night too early: ‘One (carer) came at half past 3 in the afternoon!’ Unsurprisingly, exercise and nutrition support were not her priority. Comparatively, having money and a better education meant that older adults with reduced independence could access better care and had more expansive ideas and beliefs of how to manage their health. For instance, when free intermediate care ceased, economically stable participants were able to afford healthy food delivery services and private social care to meet their needs.

#### 3.8.4 Main theme: exercise is medicine but it hurts

This theme captures older adults’ conflict between wanting to rest and avoid exercise discomfort yet believing exercise ‘is medicine’ improving health and wellbeing and preventing decline. Positive expectations, learnt from previous exercise experiences, health practitioner advise, and rehabilitation classes, motivated exercise participation. Despite the dominant belief that ‘exercise is medicine’ older adults with frailty found exercise uncomfortable, especially after deconditioning caused by illness and a hospital stay. Fatigue and pain were often reported as barriers to exercise. The discomfort and effort required to exercise led to reduced confidence and joy to move in later life. Participants highlighted the importance of pacing daily activities to suit their frail older bodies.

### 3.9 Mechanisms

#### 3.9.1 Main theme: training and volunteer support was key to safe and effective implementation

This theme captures the importance of volunteer training and support to enable volunteers to implement the intervention safely and effectively while also ensuring volunteers felt supported in their role.

Subthemes:

1. *‘Whenever I didn’t know what to say, she’d always be there’ – inexperienced volunteers needed practical and emotional support*

Volunteers were students with no previous experiences supporting older adults with frailty, so they required support from the trainer to overcome anxiety, and to ensure they flourished in their role. Volunteers illustrated challenges to process and act on difficult emotional conversations and explained the importance of the trainer’s guidance. The volunteers stayed within their role and participants needing specialist advise were handed over to the trainer. Volunteers got support from other volunteers on the programme, in which they felt more confident working together, allowing shared responsibilities. Most volunteers felt their experience could have been improved through establishing better communication amongst themselves, such as a volunteer WhatsApp group.

2. *Training structure and resources shaped volunteer expectations and prepared them for their role*

Volunteers enjoyed training which prepared them adequately for delivering the intervention. Volunteers approved of the training booklets, the training structure, found shadowing other volunteers, and role play helpful. Though the main training was acceptable, most volunteers found the recruitment process and basic volunteer checks a difficult time-consuming process.

#### 3.9.2 Main theme: the exercise intervention was acceptable for older adults without complex needs

Participants enjoyed the whole-body strengthening exercise and viewed the 12-week programme as a suitable length to gain support. The booklet was a useful addition to volunteer support, and the sessions enabled participants to effectively learn the exercise routine and boost independence. Participants who did not identify as ‘frail’ and had a familiarity with more complicated exercise found the seated exercise too easy. Comparatively, participants further along the frailty trajectory found the exercises challenging and those with poor exercise confidence viewed the support sessions as a valuable introduction to exercise training.

Despite the overall usefulness of exercises after a hospital stay, the programme was unable to specially tailor to individuals. The seated exercises were unable to fully cater for variations in ability, and some participants were concerned that some of the exercises were inappropriate for specific health conditions. Moreover, participants and volunteers would have liked the addition of standing exercise to progress mobility.

#### 3.9.3 Main theme: A hybrid approach including telephone and online support would be beneficial

This theme illustrates the advantages and disadvantages to online and telephone support and highlights that a combination of both would be more efficacious. Both forms of remote support were reported as a convenient option to access post hospital discharge, and allowed emotional support and reassurance, monitoring exercise and nutrition progress. However, volunteers felt they had the opportunity to chat more about participants’ lives and personal goals over the telephone, while facilitating group conversations online, particularly around sensitive nutrition topics, was challenging.

Exercising online ensured correct exercise technique, while monitoring exercise fidelity over the telephone was difficult. The telephone support lacked social interactions and group belonging, and the onus was on the participant to schedule in their exercise sessions. Despite depth of conversations over the telephone, the volunteers felt they built a better rapport with participants online, as they could see each other and they had more opportunities to chat, given the higher weekly frequency of these sessions compared to calls. Volunteers felt a combination of online and telephone support would be beneficial in future interventions.

#### 3.9.4 Main theme: nutrition guidance was useful but lacked depth in response to a complex nutrition landscape

Using the Nutrition Wheel, undernutrition was targeted as a conversation starter. Volunteers explained the tool was easy to use, and helped signpost participants to appropriate nutrition advice, for example, participants fortified their meals to increase calorie and protein intake. The nutrition leaflets were useful, however participants sometimes struggled to read through the leaflets comprehensively and retain the information. Most participants expected more nutrition conversations, which appeared to reduce after implementation of the Nutrition Wheel.

Participants indicated a desire for detailed nutrition discussions rather than signposting to leaflets. This presents challenges in response to the cultural shifts in food availability and messages that complicated participants’ eating behaviours. For instance, participants learnt what to eat, primarily from their families. Other participants showed that traditional diets could contradict new eating guidelines. Participants highlighted confusing information from multiple ‘fad’ diets in the media, and perceived obesity as the biggest problem, with some participants tailoring their dietary intake to prevent obesity rather than targeting nutrition to prevent or manage undernutrition and frailty.

#### 3.9.5 Main theme: supported and encouraged by the people who care helps overcome adversity

Social support and encouragement were key to motivating engagement with the programme.

Subthemes:

1. *Volunteers were caring and joyful*

A key component of the intervention was the support provided by caring, and joyful volunteers. Participants described volunteers as well mannered, cheerful, and enthusiastic, providing non-judgmental support and encouragement. Volunteers’ positive emotions transferred to participants and helped to instil confidence in older people’s lives. Volunteers indicated how they empathised with participants’ emotional struggles, listening to older adults’ illness stories, and providing a ‘sounding board’ for participants to open-up and express their feelings.

2. *Online group support was like a virtual cuddle*

The online group provided mutual support among older adults with frailty, reduced loneliness, provided a safe space to share illness stories, and created a positive vicarious experience motivating increased exercise effort. Participants felt a sense of belonging in the group and enjoyed sharing, reassuring, and learning from others. Evidence of group relatedness was expressed by the volunteers and participants, who highlighted the importance of social interactions in the group, and was an important tool to reduce social isolation, especially for older adults who struggled to leave their homes.

3. *Surrounded by friends and family’s love was vital to recovery*

Older adults with close support from family and friends were encouraged to participate in the programme and had extra care to exercise and eat well. For participants receiving telephone support, family input was helpful to bolster participation and provide direction with exercise. Family and friends were also important social resources to aid appropriate nutritional intake, helping with shopping, cooking, and general encouragement to eat when participants were struggling with suppressed appetite.

#### 3.9.6 Main theme: digital coaching and access swayed how support was received

Participants’ digital knowledge, availability of digital support, and perceptions of safety using online technology, influenced how they wanted to receive support. Age was often perceived as a restriction to using digital technology and participants indicated fear of artificial intelligence and scamming. Help from family improved digital access, for example, setting up and using Zoom. Most participants who opted for online sessions were familiar with using digital platforms, however, availability of iPads and digital coaching from the research team was useful for participants with poor digital literacy.

### 3.10 Outcomes

#### 3.10.1 Main theme: intergenerational understanding – working with older adults helped transform young volunteers’ perspectives

Inexperienced volunteers were positively pushed outside their comfort zones, opening to older adults’ difficult illness stories, which improved their understanding of participants’ everyday struggles, and gave them a sense of reward through helping others. For example, volunteers helped older adults to reappraise negative ageing stereotypes of decline. Volunteers enjoyed learning new skills, and they flourished with the responsibility of leading support sessions, enhancing their confidence to communicate and support older peoples’ health, which has inspired future careers.

#### 3.10.2 Main theme: presence of beneficial change influenced long-term activity

Participants who reported beneficial changes from exercising on the programme, such as enhanced physical health and functioning (e.g., improved strength and reduced joint stiffness) were motivated to continue exercising post-intervention and had built their exercise knowledge through the intervention to facilitate an independent exercise routine. Comparatively, participants who were further along the frailty trajectory generally did not observe any beneficial changes, which was disappointing for individuals desperate to return to cherished activities and made them question the point of exercising.

#### 3.10.3 Main theme: ‘I’ve got a bit more meat on my bones’ – improved weight and appetite

For participants with undernutrition, most noticed gradual improvements in appetite and increased weight since hospital discharge and during the programme. Acute illness often led to weight loss, in which participants grappled with complex and multifaceted influences on their nutritional status. Resolution of illness symptoms, and discharge from hospital improved appetite and some participants reported feeling hungry after exercise. Enhanced strength enabled participants to complete their own shopping, increasing their interest in planning meals.

## 4 Discussion

This study demonstrated that it is feasible and safe to implement a remote multimodal intervention, delivered by volunteers, to older adults with frailty after discharge from hospital. The intervention was acceptable to both volunteers and participants who had sufficient care to meet their basic needs. Volunteers provided emotional support, helping older adults to process their illness stories, and encouraged positive health behaviour change. Volunteers were motivated, with 5 volunteers delivering 45 online sessions and 90 telephone calls. The interviews provided greater insights into the work of the volunteers, showing that volunteers were caring and joyful, which corroborates their commitment and motivation in undertaking their role.

A key component of success was volunteer training and support through the backing of a health practitioner, especially given some of the complex needs of older adults with frailty transitioning from hospital to home. On average, each volunteer required 5.5 h of training to be signed off as competent. The training programme delivered was well received as reflected in the interviews where volunteers felt well-prepared and supported in their role. Digital coaching facilitated online access, while providing a choice between online and telephone support enabled inclusion of participants who were unable or unwilling to use technology. The online group provided a positive vicarious experience, creating a sense of belonging, while telephone calls provided reassurance and monitoring to socially isolated older adults, and an opportunity to discuss health topics in more depth.

### 4.1 Factors influencing intervention feasibility and acceptability

This study builds on existing research that shows appropriately trained volunteers can deliver exercise and nutrition support to older adults [16,17,19,26], and highlights the value of volunteers to provide social support alongside the expertise of a health professional. A central role of volunteers, in this study and others, is their ability to forge social connections and act as peer support to motivate older adults and improve confidence to engage with exercise and nutrition improvements [19,26]. In line with previous research, the online group was an important social resource, in which participants gained insight into exercise and nutrition behaviours from each other [47] and a sense of belonging with individuals experiencing comparable illness/recovery stories [48].

Challenges to engagement were unmet care needs and exercise discomfort. Increased demand and pressures on a restricted workforce, causing staff stress, exhaustion, and increased staff turnover, has affected the quality of care delivered, and the availability of trained personnel [49,50]. This context affected intervention feasibility and acceptability, indicating growing inequalities for socially isolated older adults with frailty from a lower socioeconomic background. Participants valued ‘exercise as medicine’ but struggled with the effort required to move a frail, sometimes painful older body. Emerging evidence advises health professionals to move beyond a purely ‘restitution narrative’ (i.e., exercise as a cure) to consider wellbeing, exercise enjoyment, and the personal meaning attached to PA [51,52]. Practitioners need to reinforce realistic exercise expectations and to look beyond physical adaptations to some of the immediate social and psychological advantages, such as finding an exercise they enjoy and starting with a selection of exercise considered ‘comfortable’. This is in line with emerging research that demonstrated the importance of targeting pleasure on exercise-intensity [53].

Using the Nutrition Wheel, volunteers identified 39 % of older people at risk of malnutrition (as undernutrition). Our findings extend early evaluation work, which highlighted the Nutrition Wheel as an effective tool to identify and guide older adults ‘at risk’ of undernutrition to appropriate advice and signposting [33,54,55]. Volunteers found the tool easy to use and valuable to start nutrition conversations, particularly one-to-one on the telephone rather than in an online group setting, where participants felt they had less opportunity to elaborate on sensitive nutrition topics. The utility of the Nutrition Wheel adds to a burgeoning evidence-base showing the value of volunteer-led methods in supporting adherence to a tailored nutrition guidance and intervention, such as dietary recommendations and recipes delivered through volunteers in the community [56], and improved lunchtime energy intake of older adults in hospital through mealtime assistants [16].

Participants reporting difficulties accessing services outside home, made provision of remote support options essential. Forty seven percent completed the intervention online and 53 % received telephone support. Despite the reinforcement of online interventions during and post COVID [57,58], our findings indicate the need to provide additional forms of implementation for older adults who are resistant to learning how to navigate online software and to prevent digital exclusion [58]. Reasons for preference towards telephone support included a fear of scamming, and poor confidence learning new technological skills at an older age; akin to digital barriers reported in previous works [59]. Addressing digital literacy and the appropriateness of technological interfaces for older adults [14,59], including options for co-design, may improve feasibility of future interventions [60].

Ongoing trainer support played an important role in volunteer welfare and retention, a key factor affecting intervention implementation. Young volunteers, in particular, found facilitating conversation challenging and benefitted from the trainer’s support in preparing for intergenerational interactions, including developing empathy - consistent with previous interventions [61]. While sharing difficult illness stories was emotionally challenging for younger volunteers, they were appreciative of widening their perspectives and understanding of older adults living with frailty. This study adds to existing research exploring strategies to recruit, train, and retain volunteers involved in supporting older adults’ health and wellbeing [19,26,62]. It also adds evidence of the positive impact of volunteer roles within health and social care (e.g., diversifying the workforce) [24], and the positive impact on volunteers’ confidence, upskilling, and experiences for career development [19,23].

Key aspects to consider for the scalability and sustainability of the volunteer-led intervention are the presence of financial, material, and staff resources to recruit, train, support, and retain volunteers long-term. In the current study dedicated hospital voluntary services were well established within the NHS trust and liaised closely with the research team. National policy is pushing for the development of a strategic approach to volunteering in NHS trusts [63], however, the scope of volunteering services and support differs across hospital contexts, indicating the need for further volunteer-led feasibility studies across differing hospital regions [23]. Moreover, the presence of a central trainer/supervisor is essential to provide role specific training and support, and the identification and buy-in of health practitioners to fulfil this role is a key component to scale-up the intervention.

Who volunteers are, including their qualities, also needs to be considered. While volunteer recruitment was open to anyone 16 years or older, our project attracted student volunteers, with 2 dropping out due to studying commitments. The qualities of volunteers in the current study facilitated the success of the intervention, including their capacity to deliver non-judgmental, motivational support and care to older people living with frailty. The feasibility of student volunteers in supporting a healthcare intervention for older people is consistent with previous research showing the acceptability of students as mobility volunteers on medicine for older people wards [26]. Nevertheless, consideration of the input required from a health practitioner to support training needs and ongoing support is essential for volunteer and participant safety. In other research, older people who were long-established volunteers in community social clubs were recruited to deliver a volunteer-led exercise programme to club members. The volunteers had developed a meaningful role and relationship within the clubs and they were a similar age with familiar lived experiences to the club members, hence a key quality of these volunteers was that they were relatable to participants, and became positive exercise role models [19]. In other research, and consistent with our findings, intergenerational projects provided reciprocal benefits for both young and old participants [64,65]. For instance, intergenerational volunteering in long-term care benefitted younger individuals through improving attitudes toward older adults and developing career-related skills, and benefitted care home residents through enhancing well-being, and improving engagement in care-home activities [64]. Moreover, an ‘intergenerational exercise buddy programme’ found cross-generational interactions helped motivate older people to sustain participation in community exercise classes (e.g., yoga and tai chi) [65], with similar findings reflected in the current study, in which the positivity and energy of the young volunteers transferred to older people living with frailty and helped motivate their health behaviour change.

Establishing links with the local community, such as visits to colleges, church groups, or older peoples’ social groups, and advertising across various social media outlets could help reach a diverse population of volunteers and enhance sustainability of volunteer roles. Setting out volunteer expectations at recruitment is also important to improve sustainability, such as setting eligibility criteria for age and time commitments, ensuring commitment is proportionate to recruitment and training processes and is cost-efficient. Future research should explore the feasibility and acceptability of different volunteer groups (e.g., life experiences, ages, backgrounds) to deliver a complex multimodal intervention to older people with frailty. While the Frail2fit project aligns with some of the aims of reablement services to increase function, and increase or maintain independence in a person's place of residence [10], the volunteer-led initiative is not a replacement for existing age care services. With these considerations in mind, volunteer-led projects, such as Frail2fit could work as a beneficial adjunct to reablement and as a longer-term solution to continued maintenance of health behaviour change, including exercise and eating well for older people with frailty after intermediate care ceases [11]. Key findings from this study, including social connection and encouragement from volunteers, the need to consider digital literacy and digital support for older people with frailty, and the implementation of training and ongoing volunteer support from a health practitioner, will inform refinements to the intervention for future trial designs and scalability.

### 4.2 Secondary outcomes

This feasibility study found significant improvements in self-reported TPA, appetite, and QOL post-intervention, nevertheless, our sample size was small, and these preliminary findings require confirmation in a larger controlled trial to improve generalisability. Baseline PA (PASE score 32.8) of older adults with frailty was low compared to healthy older adults in previous research (PASE score 118.9) [66], and consistent with PA levels of older adults with sarcopenia (PASE 40.2) [67]. MVPA increased from 19 to 23 min∙day^-1^, equating to 161 min∙week^-1^ - an activity level consistent with PA recommendations to improve health of older adults (i.e., 150 min of moderate, or 75 min of vigorous PA/week) [34]. Higher MVPA has been shown to significantly reduce the incidence of functional disability regardless of frailty in an inverse non-linear dose-response relationship [68]. In the current intervention, social support and exercise monitoring provided by volunteers and showcased in our theme ‘*Supported and encouraged by the people who care helps overcome adversity’,* possibly contributed to improved PA levels, with participants reporting the value of volunteers’ enthusiasm and non-judgmental support on their motivation to engage with the health programme. Moreover, the positive vicarious experience of the online group is consistent with existing literature illustrating the importance of peer role models on enhanced exercise self-efficacy (i.e., confidence in abilities to perform exercise) [19,69], and increased exercise effort through participating in a group of individuals with a similar age and functional ability [70]. Despite favourable PA levels post-intervention, rates of inactivity were high across all time points (12.2–12.5 h∙day^-1^), associated with increased risk of mortality in older people with frailty independent of PA levels [71]. Consequently, a promising direction for future research is incorporating strategies to interrupt sitting time of older adults with frailty [71], such as encouraging brief bouts of daily functional training [72]. Appetite assessment showed 41 % of participants had low baseline appetite, associated with age-related anorexia and malnutrition (SNAQ <14) [73,74]. Appetite loss in older people is common, with complex and multifaceted influences, including physiological signalling, hedonism, and environmental and societal influences [74]. Consistent with our findings, previous research showed improvements in appetite over time post-hospitalisation, with a decreased appetite reported by 51 % of older adults at hospital admission, 34 % at discharge, 28 % one month post-discharge, and 17 % three months post-discharge [75]. The observed improvements in appetite agree with our theme *‘I’ve got a bit more meat on my bones’ – improved weight and appetite’*, and suggest that resolution of illness symptoms, and enhanced ability to obtain and prepare food likely contributed to improved eating behaviours. The influence of the Nutrition Wheel on appetite is less certain, however, those receiving tailored nutrition signposting from the Nutrition Wheel indicated improved awareness of malnutrition and efforts to fortify their meals to increase energy and nutrient intake.

The secondary health improvements in our study are promising but need validation against a control group in a future randomised controlled trial, especially considering the ‘expected’ improvement in appetite, QOL, and PA post-hospitalisation. Although participant recruitment strategies were designed to reduce selection bias, there is also the possibility that participants included in the trial were more motivated to receive health support, and thus more likely to experience improvements.

### 4.3 Strengths and limitations

The time needed to complete volunteer background checks delayed completion of training and competencies, resulting in a smaller volunteer pool at the start of the intervention, which reduced session availability and resulted in a change to the tapered nature of the intervention reported in our protocol paper [32]. Once more volunteers were trained our offer increased to 3 sessions/week. This variable frequency and heterogeneity in exercise stimulus and nutrition guidance may have impacted outcomes.

The exclusion of older adults who were unable to provide consent, a small sample size, and the lack of a control group may also limit the generalisability of findings. However, using a mixed methods study design was well suited to meet our primary aim to determine the feasibility and acceptability of the intervention. Combining in-depth qualitative explorations with quantitative data, enabled detailed reporting of older adults' experiences, provided detailed insight into implementation processes, as well as examination of quantitative feasibility assessments and secondary outcome measures, which will inform larger trials. Although self-reported secondary outcome measures have provided important insights into the potential health impacts of the volunteer-led intervention, objective measures, such as the short physical performance battery to assess physical function, and anthropometric parameters to assess changes in nutritional status, should be considered in a future RCT.

## 5 Conclusions

With adequate training and healthcare practitioner support, it was safe and feasible for volunteers to deliver a remote multimodal intervention to older adults with frailty following hospital discharge. The intervention was acceptable to both volunteers and participants who had sufficient social care. The online group fostered a positive vicarious experience, and telephone calls provided reassurance and monitoring to older adults with frailty. When implementing volunteer-led interventions into routine practice, healthcare systems should consider, 1) closely liaising with hospital voluntary services to optimise volunteer recruitment and support, 2) employing a key healthcare practitioner/supervisor to train and guide volunteers, and 3) consider the qualities volunteers will need to successfully deliver the intervention (e.g., emotional intelligence). The feasibility and acceptability of volunteer-led multimodal interventions for older people with frailty needs to be explored across differing hospital contexts and regions, and future controlled trials are needed to examine the intervention’s effects on the health outcomes of this population.

## Funding

The University Hospital Southampton Foundation Trust Small Grants Scheme funded this research (Reference number: GNT0525). MG is in part funded by the NIHR Southampton Biomedical Research Centre and in part by the NIHR Senior Investigator scheme.

## CRediT authorship contribution statement

**SJ Meredith:** Investigation, Conceptualization, Writing – original draft, Formal analysis, Methodology, Data curation. **L Holt:** Investigation, Writing – review & editing. **J Varkonyi-Sepp:** Writing – review & editing, Conceptualization. **A Bates:** Writing – review & editing, Conceptualization. **KA Mackintosh:** Formal analysis. **MA McNarry:** Formal analysis. **S Jack:** Conceptualization. **J Murphy:** Conceptualization, Writing – review & editing. **MPW Grocott:** Writing – review & editing, Conceptualization. **SER Lim:** Writing – review & editing, Investigation, Supervision, Funding acquisition, Methodology, Conceptualization.

## Declaration of competing interest

The authors declare the following financial interests/personal relationships which may be considered as potential competing interests:

Samantha Jane Meredith reports financial support was provided by The University Hospital Southampton NHS Foundation Trust. Samantha Jane Meredith reports a relationship with University of Southampton that includes: employment. If there are other authors, they declare that they have no known competing financial interests or personal relationships that could have appeared to influence the work reported in this paper.

## Appendix Supplementary materials

Image, application 1
