# Supplementary material for: Frail2Fit study: it was feasible and acceptable for volunteers to deliver a remote health intervention to older adults with frailty
Source: J Frailty Aging. 2025 Oct 3;14(6):100092. doi: 10.1016/j.tjfa.2025.100092 (PMC12519248; doi:10.1016/j.tjfa.2025.100092)
Supplement: Supplementary file 1 [file mmc1.doc]

| **Skill / Knowledge**  **Frail2Fit Volunteer Competencies**  These competencies have been developed to enhance participants’ care during online sessions. They have been broken down into separate competencies to ensure you feel equipped to work with older adults to promote their activity and nutrition, whilst maintaining the individuals and your own safety. Each box should be dated and signed by yourself and the supporting therapist or researcher.  **Name: Date started: Date completed:** | **Criteria** | **Training given** | **Skill practiced with supervision on peers** | **Skill practiced with supervision on participant/group** | **Skill completed (signed by therapist / researcher)** |
| --- | --- | --- | --- | --- | --- |
| Personal safety awareness | Wear appropriate clothing and footwear to be comfortable when exercising. |  |  |  |  |
|  | Ensure your exercise space is set up safely, including a sturdy chair, and the space is free of obstacles. |  |  |  |  |
|  | Ensure you are well enough to exercise and know when exercise should be avoided. |  |  |  |  |
|  |  |  |  |  |  |
| Basic participant safety | Demonstrate what to do if a participant feels unwell. Stop exercise. Participants should not exercise if they have a fever or any new or worsening symptoms. |  |  |  |  |
|  | If participants have symptoms indicative of an emergency situation e.g., chest pain, dizziness, or shortness of breath that does not go away upon cessation of exercise, then call 999. |  |  |  |  |
|  | Understanding of the safety escalation process |  |  |  |  |
|  | Demonstrate what to do if a participant experiences pain during exercise. Stop and review the participants position and technique. Offer an alternative exercise if pain persists, or suggest they miss out the exercise and rest. Advise they seek advice from their GP if pain persists. |  |  |  |  |
|  |  |  |  |  |  |
| Pre exercise checks | Demonstrate proficiency with online set up and use of an online platform (e.g., Zoom). |  |  |  |  |
|  | Introduction to participants / group.  Explain role and overview of exercise session. |  |  |  |  |
|  | Determine participants readiness and safety to exercise. How are you? How are you feeling? Understand pre-session screening |  |  |  |  |
|  | Ensure participants have a safe exercise set-up, including a sturdy chair, water nearby for hydration, and the room is clear of obstacles. |  |  |  |  |
|  | Ensure participant has appropriate clothing and footwear to exercise. |  |  |  |  |
|  |  |  |  |  |  |
| Exercise Delivery | To demonstrate an understanding of the main components of an exercise session, including warm up, conditioning, and cool down. |  |  |  |  |
|  | To prompt and demonstrate correct technique for lower limb chair exercises. |  |  |  |  |
|  | To prompt and demonstrate correct technique for upper limb chair exercises. |  |  |  |  |
|  | To demonstrate verbal coaching proficiency, including clear feedback on exercise technique and rationale to participants. |  |  |  |  |
|  | To demonstrate ability to encourage and motivate participants to exercise. |  |  |  |  |
|  | To demonstrate understanding of exercise progressions. |  |  |  |  |
|  | | | | | |
| Nutrition Conversations | Demonstrate understanding and delivery of the nutrition wheel |  |  |  |  |
|  | To have access to nutrition resources to help signpost participants to relevant information |  |  |  |  |
|  | To understand and feel comfortable facilitating weekly nutrition topics (as listed in volunteer booklet) |  |  |  |  |
|  |  |  |  |  |  |
| Healthy Conversation Skills | Completion of online HCS training |  |  |  |  |
|  | To demonstrate understanding of goal setting and proficiency in facilitating conversations around SMARTER goals |  |  |  |  |
|  |  |  |  |  |  |
| Documentation | Proficiency completing participant registers. |  |  |  |  |
|  | Proficiency completing session completion log |  |  |  |  |
|  | Proficiency completing adverse events log. |  |  |  |  |
